# Social Determinants of Health and Insurance Claim Denials for Preventive Care

**DOI:** 10.1001/jamanetworkopen.2024.33316

**Published:** 2024-09-18

**Authors:** Alex Hoagland, Olivia Yu, Michal Horný

**Affiliations:** 1 Institute of Health Policy, Management, and Evaluation, University of Toronto, Toronto, Ontario, Canada; 2 Department of Economics, University of Toronto, Toronto, Ontario, Canada; 3 Department of Radiology and Imaging Sciences, School of Medicine, Emory University, Atlanta, Georgia; 4 Department of Health Policy and Management, Rollins School of Public Health, Emory University, Atlanta, Georgia

## Abstract

**Importance:**

The Patient Protection and Affordable Care Act (ACA) eliminated out-of-pocket cost-sharing for recommended preventive care for most privately insured patients. However, patients seeking preventive care continue to face cost-sharing and administrative hurdles, including claim denials, which may exacerbate inequitable access to care.

**Objective:**

To determine whether patient demographics and social determinants of health are associated with denials of insurance claims for preventive care.

**Design, Setting, and Participants:**

This cohort study of patients insured through their employers or the ACA Marketplaces used claims and remittance data from Symphony Health Solutions’ Integrated DataVerse from 2017 to 2020; analysis was completed from January to July 2024.

**Exposure:**

Seeking preventive care.

**Main Outcomes and Measures:**

The primary outcome was the frequency of insurer denials for preventive services across 5 categories: specific benefit denials, billing errors, coverage lapses, inadequate coverage, and other. Subgroup analysis was performed across patient household income, education, and race and ethnicity. Secondary outcomes included charges for denied claims, approximating patients’ remaining financial responsibility for care.

**Results:**

A total of 1 535 181 patients received 4 218 512 preventive services in 2 507 943 unique visits (mean [SD] age at visits, 54.02 [13.19] years; 1 804 637 visits for female patients [71.96%]); 585 299 patients (23.30%) had an annual household income $100 000 or higher, and 824 540 patients had some college education (32.88%). A total of 20 658 individuals (0.82%) were Asian, 139 950 (5.58%) were Hispanic, 219 646 (8.76%) were non-Hispanic Black, 1 372 223 (54.72%) were non-Hispanic White, and 25 412 (1.0%1) were other races and ethnicities not included in the other 4 groups. Of preventive claims, 1.34% (95% CI, 1.32%-1.36%) were denied, consisting mainly of specific benefit denials (0.67%; 95% CI, 0.66%-0.68%) and billing errors (0.51%; 95% CI, 0.50%-0.52%). The lowest-income patients had 43.0% higher odds of experiencing a denial than the highest-income patients (odds ratio, 1.43; 95% CI, 1.37-1.50; *P* < .001). The least educated enrollees had a denial rate of 1.79% (95% CI, 1.76%-1.82%) compared with 1.14% (95% CI, 1.12%-1.16%) for enrollees with college degrees. Denial rates for Asian (2.72%; 95% CI, 2.55%-2.90%), Hispanic (2.44%; 95% CI, 2.38%-2.50%), and non-Hispanic Black (2.04%; 95% CI, 1.99%-2.08%) patients were significantly higher than those for non-Hispanic White patients (1.13%; 95% CI, 1.12%-1.15%).

**Conclusions and Relevance:**

In this cohort study of 1 535 181 patients seeking preventive care, denials of insurance claims for preventive care were disproportionately more common among at-risk patient populations. This administrative burden potentially perpetuates inequitable access to high-value health care.

## Key Points

**Question:**

What is the association between patient demographics and insurance denials for preventive care among privately insured patients in the US, and which denials underlie this association?

**Findings:**

In this cohort study of 1 535 181 patients seeking preventive care, at-risk populations, including low-income patients, patients with a high school degree or less, and patients from minoritized racial and ethnic groups, experienced higher rates of claim denials. The most frequent denials were noncovered service–diagnosis code pairs and billing errors.

**Meaning:**

These findings suggest that experiences of patients seeking free preventive care differ on the basis of their demographics, leading to inequities in accessing basic preventive care.

## Teaser

This cohort study investigates whether patient demographics and social determinants of health are associated with denials of insurance claims for preventive care.

## Introduction

Exposure to cost-sharing for health care reduces future use, regardless of whether a service is high value, such as preventive care.^1,2,3^ Although the Patient Protection and Affordable Care Act (ACA) exempted many high-value preventive services from cost-sharing,^4^ many patients continue to pay out-of-pocket (OOP) for these services because of errors or misunderstandings among patients, hospitals, billing staff at a medical institutions, and insurers.^5,6^ Commonly denied preventive services, including high-value cancer clinical trials associated with detection and reproductive health care, generate substantial cost-sharing for patients.^7,8,9^ Patients continually report that the affordability of health care is a primary concern, with 74% of adults in 2024 expressing that they are very or somewhat worried about affording unexpected medical bills.^10^ These unexpected bills similarly discourage households from using future medical care.^11^

Unanticipated cost-sharing for preventive care has important implications for equitable access to high-value services. Use of preventive care persists at well below recommended rates,^12,13^ and take-up is lower for marginalized and at-risk patient groups, including socioeconomically disadvantaged individuals, patients with little formal education, and individuals from minoritized racial and ethnic groups.^14,15,16,17,18^ Cost-sharing exacerbates these disparities given patient sensitivity to OOP costs.^19,20,21^ However, little is known about how patient demographics and social determinants of health (SDOH) are associated with claims denials within an insured population.

Associations between SDOH and denials may be the result of multiple factors. If patients of different groups select different levels of insurance coverage with limited administrative resources (eg, low-income patients purchasing lower-generosity coverage), they may experience denials more frequently.^22,23^ In addition, patients may seek preventive care from different health care organizations with different billing practices or propensities for denials. Finally, factors such as language and communication barriers or systemic discrimination may contribute to different denial rates.

In this study, we examine the association of patient demographics and SDOH—including income, education, and race and ethnicity—with claim denial rates for preventive care, using a large, national sample of patients in all 50 US states enrolled in employer-sponsored insurance (ESI) or ACA Marketplace plans between 2017 and 2020. Importantly, our data source allows us to identify some of the stated causes of denials and residual cost-sharing,^24^ which may differentially leave patients at risk of inappropriate cost-sharing.

## Methods

### Research Design and Study Sample

This cohort study was based on deidentified proprietary data and did not constitute human participants research as defined by 45 CFR §46.102; hence, it was exempted by the University of Toronto Research Ethics Board, and informed consent was not sought. We followed the Strengthening the Reporting of Observational Studies in Epidemiology (STROBE) reporting guidelines.^25^

We used data from Symphony Health Solutions’ Integrated Dataverse from 2017 to 2020.^26^ The data contained information on patients, including demographics, as well as detailed claims and remittance information for health care encounters. The data spanned patients from multiple insurance payers in all 50 US states and the District of Columbia, including patients enrolled in both ESI and ACA Marketplace plans. We restricted the data to include only patients aged 18 to 65 years who were observed continuously for at least 6 months.

SDOH data in the Symphony Health Solutions included self-reported information on household income, education, and race and ethnicity. These data were compiled from sources such as purchase transactions and voter registration, and then were linked to claims data. Race and ethnicity data were classified using self-reported and electronic health record data, and then were enhanced by the data provider using an algorithm leveraging patient name and geographic location. Race and ethnicity categories included Asian, Hispanic, non-Hispanic Black and African American, non-Hispanic White and Caucasian, and other, which included any individual unidentified by at least 1 of the 4 groups. Demographic data were included for approximately 70% of observed enrollees. Previous work^27,28^ has used these demographic and SDOH data when studying patient outcomes and health equity.

For each enrollee, we identified the use of 7 preventive services recommended by the US Preventive Services Task Force that would have been subject to the ACA preventive care provision. Services included contraceptive administration, breast cancer screening, cholesterol screening, colorectal cancer screening, depression screening, diabetes screening, and wellness visits. *Current Procedural Terminology* procedure codes and *International Statistical Classification of Diseases, Tenth Revision, Clinical Modification* diagnosis codes used to identify these services are listed in eTable 1 in Supplement 1.

### Study Variables and Methods

We measured differential exposure to administrative burdens from accessing preventive care across patient demographics and SDOH. Our primary outcomes were the incidence of claim denials overall and by categories of listed reasons for denial: specific benefit denials (decided by the insurer), billing errors (influenced by physician billing practices), coverage lapses, inadequate coverage, and other reasons (eTable 2 in Supplement 1).

This categorization of denials allowed us to identify overall patterns among the stated reasons services were denied. Denials may indicate that a specific benefit was not covered under a person’s plan, that physicians did not correctly bill the insurer for a service (eg, with necessary diagnostic codes attached), that patient coverage by a specific insurer had lapsed, or that patients had multiple sources of insurance coverage, among others. Our categorization disaggregated these associations for preventive services, where certain approval processes (eg, prior authorizations) are not typically required. Claims with other stated denial reasons, such as denials that bundled payment of multiple services commonly performed together, were not included as denials in this analysis, because these only altered physician or hospital payments and did not typically expose patients to cost-sharing.

Finally, we measured the portion of a denied claim that went unpaid by an insurer as the difference between the total billed charges and insurer-paid amounts. Denied claims that were reprocessed were identified on the basis of common identifiers for patients, physicians or hospitals, procedure code, and dates; the remaining difference between the final total billed amounts and final insurer payments were aggregated at the claim level and top-coded at $250 000. Identifying resubmitted claims allowed us to determine subsequent success in processing a claim and the corresponding reductions in patient responsibility. Denied claims that were never resubmitted, however, did not have information on final payments, so these amounts do not always correspond to patient responsibility, because physicians may write off income for denied services.

### Statistical Analysis

We compared denial rates and unpaid claim amounts across patient demographics using 2-sided *t* tests with a significance threshold of *P* < .05 and multivariable logistic regression adjustment for preventive service types, patient 2-digit zip code, and insurer. We included individual-specific random effects to accommodate correlations across multiple preventive encounters. We performed analyses from January to July 2024 using Stata MP statistical software version 15 (StataCorp) and R statistical software version 4.2.0 (R Project for Statistical Computing).

## Results

Our sample consisted of 4 218 512 preventive services (10 325 569 claims) delivered to 1 535 181 patients in 2 507 943 unique visits (mean [SD] age at visits, 54.02 [13.19] years; 1 804 637 visits for female patients [71.96%]). A total of 20 658 individuals (0.82%) were Asian, 139 950 (5.58%) were Hispanic, 219 646 (8.76%) were non-Hispanic Black, 1 372 223 (54.72%) were non-Hispanic White, and 25 412 (1.0%1) were other races and ethnicities not included in the other 4 groups. In total, 585 299 individuals (23.30%) had annual household income over $100 000 USD, and 824 540 individuals (32.88%) had some college education (Table 1). Of all preventive claims 1.34% (95% CI, 1.32%-1.36%) were denied; among those incurring OOP costs, the median (IQR) bill was $630 ($286-$1165). The most common types of denials were specific benefit denials (0.67%; 95% CI, 0.66%-0.68%) and billing errors (0.51%; 95% CI, 0.50%-0.52%).

**Table 1.** Summary Statistics

Characteristic	Visits, No. (%)^a^				
	All services (N = 2 507 943)	Contraceptive services (n = 591 143)	Diabetes or cholesterol screening (n = 767 923)	Depression screening (n = 123 544)	Wellness visits (n = 1 667 311)
Patients					
Age, mean (SD), y	54.02 (13.19)	51.88 (14.56)	56.77 (12.06)	51.82 (15.31)	53.19 (13.53)
Sex					
Female	1 804 637 (71.96)	569 149 (96.28)	447 147 (58.23)	78 529 (63.56)	1 166 889 (69.99)
Male	703 306 (28.04)	21 994 (3.72)	320 776 (41.77)	45 015 (36.44)	500 422 (30.01)
Race and ethnicity					
Asian	20 658 (0.82)	3975 (0.67)	6704 (0.87)	949 (0.77)	13 096 (0.79)
Hispanic	139 950 (5.58)	27 847 (4.71)	43 741 (5.70)	8352 (6.76)	94 090 (5.64)
Non-Hispanic Black	219 646 (8.76)	55 219 (9.34)	77 855 (10.14)	9267 (7.50)	147 150 (8.83)
Non-Hispanic White	1 372 223 (54.72)	297 186 (50.27)	419 350 (54.61)	67 292 (54.47)	909 941 (54.58)
Other^b^	25 412 (1.01)	5373 (0.91)	8022 (1.04)	1202 (0.97)	16 484 (0.99)
Missing	730 054 (29.11)	201 543 (34.09)	212 251 (27.64)	36 482 (29.53)	486 550 (29.18)
Annual household income, $					
<30 000	313 972 (12.52)	86 209 (14.58)	104 947 (13.67)	14 534 (11.76)	208 859 (12.53)
30 000-49 999	250 672 (10.00)	61 982 (10.49)	85 459 (11.13)	11 550 (9.35)	168 428 (10.10)
50 000-74 999	333 707 (13.31)	76 123 (12.88)	110 045 (14.33)	15 397 (12.46)	220 801 (13.24)
75 000-99 999	320 582 (12.78)	64 990 (10.99)	100 428 (13.08)	15 738 (12.74)	211 924 (12.71)
≥100 000	585 299 (23.34)	105 266 (17.81)	162 294 (21.13)	31 331 (25.36)	388 442 (23.3)
Missing	703 711 (28.06)	196 573 (33.25)	204 750 (26.66)	34 994 (28.33)	468 857 (28.12)
Education					
High school or less	478 062 (19.06)	121 725 (20.59)	161 966 (21.09)	20 668 (16.73)	320 057 (19.20)
Some college	824 540 (32.88)	178 277 (30.16)	257 554 (33.54)	40 505 (32.79)	544 486 (32.66)
Associate degree or higher	479 867 (19.13)	89 356 (15.12)	136 562 (17.78)	26 465 (21.42)	319 140 (19.14)
Missing	725 474 (28.93)	201 785 (34.13)	211 841 (27.59)	35 906 (29.06)	483 628 (29.01)
Services^c^					
Billed cost, total (median), $	2145 (630)	4149 (748)	2552 (936)	1511 (880)	1780 (614)
Denials					
All	33 564 (1.34)	9492 (1.61)	13 854 (1.80)	3370 (2.73)	19 701 (1.18)
Benefit denial	16 895 (0.67)	6069 (1.03)	6202 (0.81)	2110 (1.71)	10 121 (0.61)
Billing error	12 786 (0.51)	2514 (0.43)	6320 (0.82)	1065 (0.86)	6907 (0.41)
Coverage lapse	3472 (0.14)	958 (0.16)	1105 (0.14)	286 (0.23)	2585 (0.16)
Alternative coverage	2526 (0.10)	669 (0.11)	896 (0.12)	119 (0.01)	1743 (0.10)
Other	568 (0.02)	168 (0.03)	248 (0.03)	101 (0.08)	359 (0.02)

^a^ Inclusion in a column requires that a patient received that specific preventive service on the included date. Sample sizes represent the total number of patient-date pairs that include the index preventive service; this includes a total of 4 218 512 preventive services.

^b^ Other race and ethnicity category includes any patient with identified race or ethnicity not in the primary 4 categories.

^c^ See eTable 1 in Supplement 1 for service definitions based on procedural and diagnostic codes. Billed cost is reported in 2024 US dollars.

**Figure 1.**
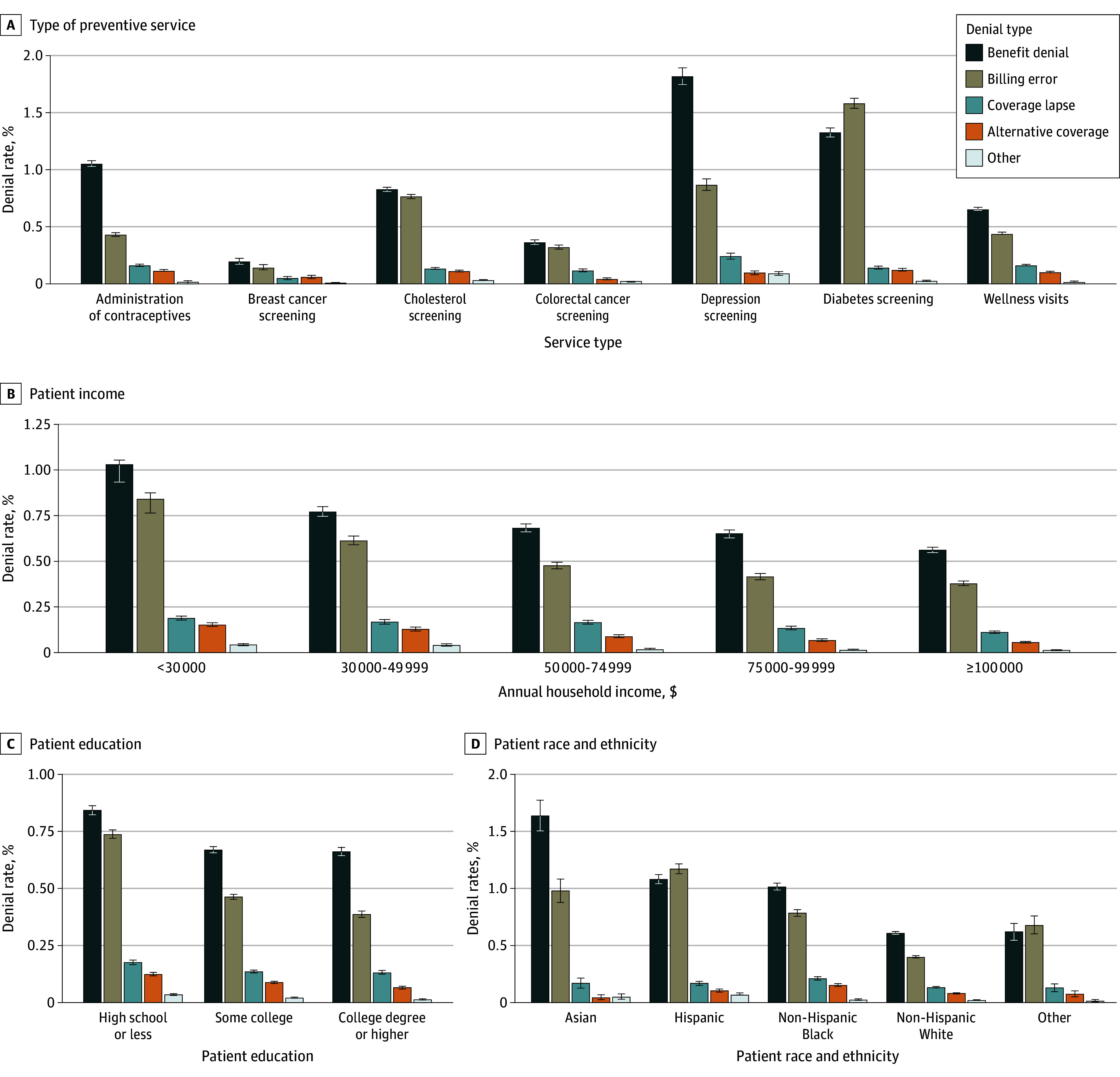
Preventive Claim Denial Rates and Patient Demographics Graphs show unadjusted means for preventive benefit denials (measured in percentages; error bars denote 95% CIs) across reported patient demographic information, including type of preventive service (eTable 1 in Supplement 1), patient household income, patient education, and patient race and ethnicity. Patients with missing demographic information are removed from each graph. Other race and ethnicity category includes any patient with identified race or ethnicity not in the primary 4 categories. Denial types are defined in eTable 2 in Supplement 1.

Denial rates differed meaningfully across preventive services and patient demographics (eFigure 1 in Supplement 1). Overall, denials were most common for diabetes screening (3.06%; 95% CI, 3.00%-3.12%), depression screening (2.84%; 95% CI, 2.75%-2.93%), cholesterol screening (1.75%; 95% CI, 1.72%-1.77%), and contraceptive administration (1.64%; 95% CI, 1.61%-1.67%) (Figure 1A). Specific benefit denials were most frequently observed for depression screening (1.82%; 95% CI, 1.75%-1.89%), contraceptive administration (1.05%; 95% CI, 1.03%-1.08%), and cholesterol screening (0.83%; 95% CI, 0.81%-0.85%), whereas billing errors constituted most denials for diabetes screening (1.58%; 95% CI, 1.54%-1.63%). Benefit denials constituted 58.8% of all denials for contraceptive administration and 59.1% of all denials for depression screening.

Significant differences in claim denials were observed according to patient demographics. Lower household income was associated with increased rates of both benefit denials and billing errors (Figure 1B). Households in the lowest income bracket (<$30 000 annually) experienced claim denials at a rate of 2.11% (95% CI, 2.07%-2.15%), whereas households in the highest income bracket (≥$100 000 annually) experienced denials 1.02% of the time (95% CI, 1.00%-1.04%). A similar gradient was observed for patient education (Figure 1C): denial rates for enrollees with a high school degree or less were 1.79% (95% CI, 1.76%-1.82%) compared with 1.14% (95% CI, 1.12%-1.16%) for enrollees with college degrees. Differences were especially large for billing errors, with the lowest-educated enrollees incurring denials at a rate of 0.74% (95% CI, 0.72%-0.76%) compared with 0.39% (95% CI, 0.37%-0.40%) for the highest educated. Finally, significant differences were observed across patient race and ethnicity (Figure 1D). Non-Hispanic White patients experienced denials at a rate of 1.13% (95% CI, 1.12%-1.15%) compared with 2.72% (95% CI, 2.55%-2.90%) for Asian patients, 2.44% (95% CI, 2.38%-2.50%) for Hispanic patients, and 2.04% (95% CI, 1.99%-2.08%) for non-Hispanic Black patients. Differences were observed for both benefit denials and billing errors, with benefit denials most common for Asian patients (1.64%; 95% CI, 1.51%-1.77%).

Regression adjustment preserved these findings for income and race and ethnicity, but not for education (Table 2). Patients in the lowest income group had 43.0% higher odds of experiencing any denial than patients in the highest income group (odds ratio, 1.43; 95% CI, 1.37-1.50; *P* < .001), with differences in the likelihood of both benefit denials and billing errors. Meanwhile, patients from minoritized racial and ethnic groups experienced significantly more denials, with odds ratios of 1.19 (95% CI, 1.15-1.24; *P* < .001) for non-Hispanic Black patients, 1.16 (95% CI, 1.10-1.12; *P* < .001) for Hispanic patients, and 1.54 (95% CI, 1.39-1.70; *P* < .001) for Asian patients compared with non-Hispanic White patients. Adjusted differences in denial rates across the lowest-educated and highest-educated patients were not statistically significant.

**Table 2.** Logistic Regression Analysis, Claim Denials

Variable	OR (95% CI)^a^		
	Any claim denial	Specific benefit denial	Billing error denial
Annual patient household income, $			
<30 000	1.43 (1.37-1.50)^b^	1.53 (1.44-1.63)^b^	1.16 (1.08-1.25)^b^
30 000-49 999	1.26 (1.20-1.33)^b^	1.28 (1.20-1.37)^b^	1.10 (1.02-1.19)^c^
50 000-74 999	1.16 (1.11-1.22)^b^	1.25 (1.17-1.33)^b^	1.00 (0.92-1.07)
75 000-99 999	1.11 (1.06-1.16)^b^	1.20 (1.12-1.27)^b^	0.99 (0.92-1.07)
≥100 000	1 [Reference]	1 [Reference]	1 [Reference]
Patient education			
High school or less	0.99 (0.95-1.04)	0.96 (0.90-1.02)	1.04 (0.97-1.12)
Some college	0.96 (0.92-0.996)^c^	0.90 (0.86-0.95)^b^	1.04 (0.97-1.11)
Associate’s degree or higher	1 [Reference]	1 [Reference]	1 [Reference]
Patient race and ethnicity			
Asian	1.54 (1.39-1.70)^b^	1.94 (1.71-2.20)^b^	1.14 (0.70-1.35)
Hispanic	1.16 (1.10-1.22)^b^	1.30 (1.21-1.40)^b^	1.02 (0.94-1.10)
Non-Hispanic Black	1.19 (1.15-1.24)^b^	1.21 (1.15-1.29)^b^	1.15 (1.08-1.22)^b^
Non-Hispanic White	1 [Reference]	1 [Reference]	1 [Reference]
Other^d^	0.89 (0.79-1.00)	0.80 (0.67-0.95)^c^	0.95 (0.80-1.13)

Abbreviation: OR, odds ratio

^a^ Regression adjustment indicates ORs from logistic regression estimating binary outcomes for any claims denial (primary outcome) and specific benefit or billing error denials (suboutcomes). Additional controls with coefficients not reported include dummy variables for service types.

^b^ *P* < .001.

^c^ *P* < .05.

^d^ Other race and ethnicity category includes any patient with identified race or ethnicity not in the primary 4 categories

**Figure 2.**
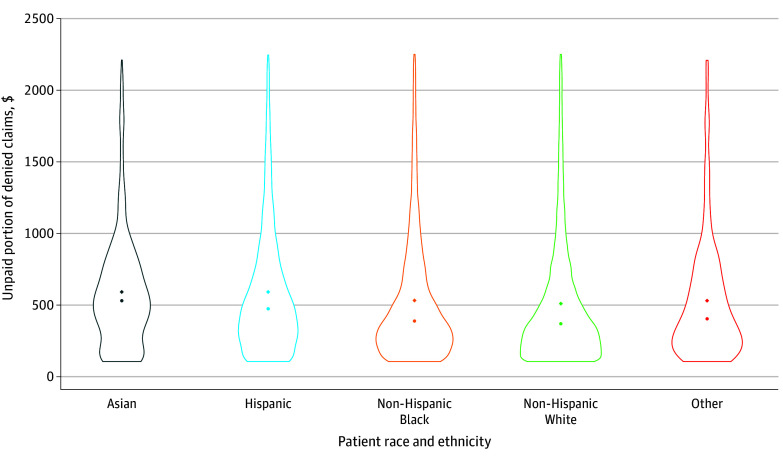
Distributions of Unpaid Portion of Denied Preventive Claims, by Race and Ethnicity Violin plots illustrate distributions for the unpaid portion of denied claims for preventive services (defined in eTable 1 in Supplement 1). Other race and ethnicity category includes any patient with identified race or ethnicity not in the primary 4 categories. Claims include preventive services as well as any additional services consumed by the patient at the same physician on a given day; claims are then aggregated to the patient-physician-service day level. The unpaid portion of a claim is measured as the total charges for the denied claim minus any amount paid by the plan and are adjusted to be in 2024 US dollars. Distributions are conditional on a denial; the graph removes patient responsibility greater than $2500 for readability. Distribution means are shown using diamonds; medians are reported using dots.

Patient demographics were also associated with differences in the amount of unpaid claims among denied services (Figure 2 and eTable 3 in Supplement 1). Only 32.4% (95% CI, 32.3%-32.5%) of denied claims were resubmitted by physicians following a denial, which resulted in denied claims having unpaid portions—presumably left to patients—92.85% of the time, with mean bills of $1395 (95% CI, $1372-$1418; median, $385). This varied across patient income and race and ethnicity, with low-income patients facing a higher burden (median, $412; 95% CI, $404-$420) than high-income patients (median, $365; 95% CI, $357-$372), and non-Hispanic Black (median, $390; 95% CI, $382-$398), Hispanic (median, $464; 95% CI, $458-$472), and Asian (median, $522; 95% CI, $512-$532) patients each facing higher costs than non-Hispanic White patients (median, $357; 95% CI, $351-$363). Smaller differences were observed across less-educated patients (median, $384; 95% CI, $376-$392) compared with those with more education (median, $399; 95% CI, $392-$406).

Results were robust to excluding all nonpreventive same-day services (eFigure 2 in Supplement 1) and wellness visits (eFigure 3 in Supplement 1). Finally, results were robust to excluding multiple claims per individual or including an individual-specific random effect to address correlated claims by the same individuals (eTable 4 in Supplement 1).

## Discussion

In this cohort study, we examined the association between patient SDOH and insurance claim denials for preventive services, which should be cost-sharing exempt under the ACA. We highlight 3 important findings. First, patients with lower incomes experienced denials more frequently than patients with higher incomes, as did those with less education or those from minoritized racial and ethnic groups. Second, differences across income and race and ethnicity remained significant even after adjusting for patient geography, service type, and insurer. Third, services for at-risk patient populations, including contraceptive administration or mental health screenings, were associated with higher denial rates than other preventive services.

Importantly, more than two-thirds of denied claims were never resubmitted by a physician. Unless these claims were written off by physicians as lost revenue, the costs of denied claims may be inequitably transferred to at-risk patients.^29^ Conditional on a denied claim, patients who were more likely to experience denials also faced higher cost-sharing.

Our findings provide evidence that health inequities exist in primary care consumption. Differences in denial rates were associated with benefit denials (ie, services deemed not covered by a plan) and billing errors (ie, billing without appropriate modifier codes^30^), which may arise for multiple reasons. Patients may sort into insurance plans with fewer administrative resources, or with higher propensities for billing errors. This sorting may occur even across different plans offered by 1 insurer. Similarly, patients may visit different health care organizations with different billing practices. In addition, our results may be explained, in part, by language and communication barriers, geographic differences in billing and processing claims (beyond those observed at the 2-digit zip code level), or systemic discrimination (potentially by algorithmic claims processing).^31^ For example, communication barriers may limit patient-physician communication for some patient groups,^32,33,34^ potentially affecting the health of some immigrant communities.^35^ If these misunderstandings manifest in differences between a patient’s understanding of their care and actual physician billing practices, these patients may be more likely to face denials. This may be particularly relevant for Asian patients, who experienced especially high rates of benefit denials.

These inequitable barriers may affect both patients’ health and future health care use. Denials and unexpected bills for supposedly free care may impact trust in the health care system, which reduces the chance a patient will pursue additional care.^36^ Importantly, observed differences may be further exacerbated if at-risk populations are less likely to appeal denials^37^; the compounded effects of inequitable distributions and differential appeals of denials may exacerbate inequities. Finally, claim denials are closely interrelated with other unexpected cost-sharing for preventive care. For example, free screening with a positive test result may not be cost-sharing exempt,^38,39^ and some arrangements between physicians and insurers generate patient costs for necessary equipment, such as surgical trays for a screening colonoscopy.^40^

Previous work^41^ has shown that denial rates differ across insurance types, but, to our knowledge, no work has quantified differences within an insured population according to SDOH. The average denial rate for Silver ACA Marketplace plans is 17.3%,^42^ with roughly 1 in 4 denials from a primary care office visit attributed to coding errors.^37,43^ Our reported denial rates are based on preventive services; hence, a reported average of approximately 1 in 60 patients is concerning. Our work is similarly related to research highlighting ordeals and administrative burdens in health care. These ordeals—including billing errors and back-and-forth among physicians, insurers, and patients—generate frictions that may cause physicians to avoid treating patients who are viewed as bureaucratically complex.^44^ Finally, our results are related to research examining prior authorization denials for Medicaid, which were concentrated among plans with especially low-income enrollees.^45^

Our findings have important policy implications. First, uniform billing standards may improve patient experiences, particularly as different patient groups have differing insurance coverage.^46^ Regulations providing clear coverage information to patients or billing guidance to physicians and payers may improve equitable adjudication across patient groups. Second, resolving differences in billing practices, including across geography, may improve patient experiences and outcomes. Third, improved communication and language assistance may mitigate some of the observed differences in denial rates across minoritized racial and ethnic groups, potentially improving equitable access to outpatient care. Finally, health equity frameworks are vital at all points of contact with the health care system, including for preventive care.^47^

### Limitations

This study had several limitations. First, we identified preventive services exclusively on the basis of billed diagnosis and procedure codes in the claims data, as in previous work. Some included claims were likely not processed as preventive (eg, many insurers cover only 1 preventive wellness visit annually), whereas some claims we excluded may have been intended as preventive (eg, screening colonoscopies without the appropriate modifier differentiating it from a diagnostic examination). There is considerable overlap in preventive coding guidelines, and our algorithm was inclusive of federal guidance and several major ESI insurers represented in our sample.^6^ In addition, our results were robust to excluding services occurring on the same day as preventive services or services prone to misclassification, such as wellness visits.

Second, although our data provided rich demographic information, they were incomplete for some patients in our sample. To the extent that missing demographic information is correlated with patients in marginalized situations (eg, those without a valid credit score or voter registration), our estimates of differences in denial rates across patient demographic groups may understate true differences, instead providing lower bounds that may be refined with future research. Future research may also isolate the unique mechanisms underlying observed differences across groups, such as the hypothesized role of language and communication barriers for some patients, or the role of appealing denials in mitigating or exacerbating inequitable outcomes.

Third, our analysis was limited to enrollees of ESI or ACA Marketplace plans. This enabled us to examine denial rate variation within an insurance type, but future research should confirm whether these results generalize to other insured populations, such as those enrolled in Medicare or Medicaid.

## Conclusions

This cohort study examined the association of patient demographics and inappropriate billing for preventive care, including claim denials and cost-sharing. Patients from at-risk groups, including those with low household incomes and little formal education and those from minoritized racial and ethnic backgrounds were more likely to have claims for preventive services denied or incur cost-sharing for these services that should be cost-sharing exempt. This study adds to the policy discussions around promoting equitable access to primary health care, including preventive services. Our findings highlight that greater attention must be paid to patient demographics when promoting policies to ensure free access to preventive care.

## Notes

Supplement 1. **eTable 1.** List of Procedure and Diagnostic Codes Used to Identify Preventive Claims **eTable 2.** List and Frequency of Claims Denials Reasons by Category **eTable 3.** Conditional Patients’ Financial Responsibility for Denied Preventive Claims **eTable 4.** Robustness of Regression Results to Individual-Specific Random Effects **eFigure 1.** Overall Denial Rates **eFigure 2.** Robustness of Inclusion of All Same-Day Services **eFigure 3.** Robustness of Exclusion of Wellness Visits

Supplement 2.
Data Sharing Statement
