# Supplementary material for: Social Determinants of Health and Insurance Claim Denials for Preventive Care
Source: JAMA Netw Open. 2024 Sep 18;7(9):e2433316. doi: 10.1001/jamanetworkopen.2024.33316 (PMC11411384; doi:10.1001/jamanetworkopen.2024.33316)
Supplement: Supplement 1. — eTable 1. List of Procedure and Diagnostic Codes Used to Identify Preventive Claims eTable 2. List and Frequency of Claims Denials Reasons by Category eTable 3. Conditional Patients’ Financial Responsibility for Denied Preventive Claims eTable 4. Robustness of Regression Results to Individual-Specific Random Effects eFigure 1. Overall Denial Rates eFigure 2. Robustness of Inclusion of All Same-Day Services eFigure 3. Robustness of Exclusion of Wellness Visits [file jamanetwopen-e2433316-s001.pdf]

Supplemental Online Content

Hoagland A, Yu O, Horný M. Social determinants of health and insurance claim denials for preventive care. *JAMA Netw Open*. 2024;7(9):e2433316.
doi:10.1001/jamanetworkopen.2024.33316

eTable 1. List of Procedure and Diagnostic Codes Used to Identify Preventive Claims

eTable 2. List and Frequency of Claims Denials Reasons by Category

eTable 3. Conditional Patients' Financial Responsibility for Denied Preventive Claims

eTable 4. Robustness of Regression Results to Individual-Specific Random Effects

eFigure 1. Overall Denial Rates

eFigure 2. Robustness of Inclusion of All Same-Day Services

eFigure 3. Robustness of Exclusion of Wellness Visits

This supplemental material has been provided by the authors to give readers additional information about their work.

© 2024 Hoagland A et al. *JAMA Network Open.*

eTable 1: List of Procedure and Diagnostic Codes Used to Identify Preventive Claims

Service Type	CPT Procedure Codes	Required Accompanying ICD-10-CM Diagnosis Codes
Administration and management of contraceptives	96372, 57170, 58340, 58565, 58600, 58605, 58611, 58615, 58670, 58671, 74740, A4261, A4264, A4266, 11976, 11981, 11982, 11983, 58300, 58301, J1050, J1051, J1055, J1056, J7302, J7306, J7307, S4981, S4989, J7300, Q0090, V2501, V2502, V2503, V2509, V2511, V2512, V2513, V2540, V2541, V2542, V2543, V2549, V255, V258, V259, Z30011, Z30012, Z30013, Z30014, Z30018, Z30019, Z3009, Z3040, Z3041, Z3042, Z30430, Z30431, Z30432, Z30433, Z3049, Z308, Z309	
Breast Cancer Screening	77057, 77052, G0202, 77067, 77065, 77066, 76083, 76092	
Cholesterol Screening	36415, 36416, 80061, 82465, 83718, 83719, 83721, 84478	Z0000, Z0001, Z13220
Colorectal Cancer Screening	44388, 44389, 44392, 44393, 44394, 45300, 45301, 45302, 45303, 45304, 45305, 45306, 45307, 45308, 45309, 45310, 45311, 45312, 45313, 45314, 45315, 45316, 45317, 45318, 45319, 45320, 45330, 45331, 45332, 45333, 45334, 45335, 45338, 45339, 45340, 45378, 45379, 45380, 45381, 45382, 45383, 45384, 45385, 45386, 74263, 82270, 82274, 88304, 88305, G0104, G0105, G0106, G0107, G0120, G0121, G0122, G0328, G0394, S0601, S3890	Z0000, Z0001, Z1210, Z1211, Z1212, Z800, Z8371, Z8379
Depression Screening	96127, 96160, 96161, 99420, G0444	Z1389
Diabetes Screening	36415, 36416, 82947, 82948, 82950, 82951, 82952, 83036	Z0000, Z0001, Z131, I10, I110, I119, I120, I129, I130, I1310, I1311, I132, I150, I151, I152, I158, I159, N262, O10011, O10012, O10013, O10019, O1002, O1003, O10111, O10112, O10113, O10119, O1012, O1013, O10211, O10212, O10213, O10219, O1022, O1023, O10311, O10312, O10313, O10319, O1032, O1033, O10411, O10412, O10413, O10419, O1042, O1043, O10911, O10912, O10913, O10919, O1092, O1093, O111, O112, O113, O119, O131, O132, O139, O161, O162, O163, O169
Wellness Visits	99201, 99202, 99203, 99204, 99205, 99211, 99212, 99213, 99214, 99215, 99381, 99382, 99383, 99384, 99388, 99389, 99390, 99391, 99392, 99393, 99394, 99401, 99402, 99403, 99404, 99411, 99412, 99461, G0101, G0344, G0402, G0438, G0439, G0445, S0610, S0612, S0613	Z0000, Z0001, Z00110, Z00111, Z00129

© 2024 Hoagland A et al. *JAMA Network Open.*

eTable 2: List and Frequency of Claims Denials Reasons by Category

1. Alternative Coverage

Code	Frequency, All Claims	Frequency, Preventive Services Only	Description
19	391	25	This is a work-related injury/illness and thus the liability of the Worker's Compensation Carrier.
20	68	3	This injury/illness is covered by the liability carrier.
21	90	5	This injury/illness is the liability of the no-fault carrier.
22	13,104	632	This care may be covered by another payer per coordination of benefits.
23	104,306	3,131	The impact of prior payer(s) adjudication including payments and/or adjustments.
24	41,021	135	Charges are covered under a capitation agreement/managed care plan.
109	3,407	454	Claim/service not covered by this payer/contractor. You must send the claim/service to the correct payer/contractor.
169	352	5	Alternate benefit has been provided.

2. Specific Benefit Denials

Code	Frequency, All Claims	Frequency, Preventive Services Only	Description
35	868	33	Lifetime benefit maximum has been reached.
39	80	67	Services denied at the time authorization/pre-certification was requested.
40	79	5	Charges do not meet qualifications for emergent/urgent care.
46	4	4	This (these) service(s) is (are) not covered.
50	3,467	2,752	These are non-covered services because this is not deemed a 'medical necessity' by the payer.
51	63	38	These are non-covered services because this is a pre-existing condition.
54	91	4	Multiple physicians/assistants are not covered in this case.
55	3,350	2,611	Procedure/treatment is deemed experimental/investigational by the payer.
56	24	22	Procedure/treatment has not been deemed 'proven to be effective' by the payer.
58	424	58	Treatment was deemed by the payer to have been rendered in an inappropriate or invalid place of service.
95	1,426	69	Plan procedures not followed.
96	43,971	2,836	Non-covered charge(s).
119	9,610	891	Benefit maximum for this time period or occurrence has been reached.
149	30	1	Lifetime benefit maximum has been reached for this service/benefit category.
150	685	46	Payer deems the information submitted does not support this level of service.
151	832	117	Payment adjusted because the payer deems the information submitted does not support this many/frequency of services.
160	435	140	Injury/illness was the result of an activity that is a benefit exclusion.
165	395	19	Referral absent or exceeded.
167	2,101	271	This (these) diagnosis(es) is (are) not covered.
170	568	46	Payment is denied when performed/billed by this type of provider.
171	154	16	Payment is denied when performed/billed by this type of provider in this type of facility.

© 2024 Hoagland A et al. *JAMA Network Open.*

172	25	21	Payment is adjusted when performed/billed by a provider of this specialty.
177	182	30	Patient has not met the required eligibility requirements.
197	5,112	1,003	Precertification/authorization/notification absent.
198	47	12	Precertification/authorization exceeded.
204	9,693	7,183	This service/equipment/drug is not covered under the patient's current benefit plan
227	9,055	400	Information requested from the patient/insured/responsible party was not provided or was insufficient/ incomplete.
231	3,067	153	Mutually exclusive procedures cannot be done in the same day/setting.
242	4,011	268	Services not provided by network/primary care providers.
256	1,282	86	Service not payable per managed care contract.
272	445	16	Coverage/program guidelines were not met.
273	386	33	Coverage/program guidelines were exceeded.
B1	1,136	47	Non-covered visits.
B15	8,996	460	This service/procedure requires that a qualifying service/procedure be received and covered. The qualifying other service/procedure has not been received/adjudicated.
B5	383	22	Coverage/program guidelines were not met or were exceeded.

3. Billing Errors

Code	Frequency, All Claims	Frequency, Preventive Services Only	Description
4	7,023	785	The procedure code is inconsistent with the modifier used or a required modifier is missing.
5	656	97	The procedure code/bill type is inconsistent with the place of service.
6	1,586	162	The procedure/revenue code is inconsistent with the patient's age.
7	48	9	The procedure/revenue code is inconsistent with the patient's gender.
8	432	6	The procedure code is inconsistent with the provider type/specialty (taxonomy).
9	129	16	The diagnosis is inconsistent with the patient's age.
10	134	11	The diagnosis is inconsistent with the patient's gender.
11	4,326	3,187	The diagnosis is inconsistent with the procedure.
12	3	3	The diagnosis is inconsistent with the provider type.
15	351	45	The authorization number is missing, invalid, or does not apply to the billed services or provider.
16	15,445	1,751	Claim/service lacks information or has submission/billing error(s) which is needed for adjudication.
29	15,100	1,946	The time limit for filing has expired.
47	8	8	This (these) diagnosis(es) is (are) not covered, missing, or are invalid.
49	3,126	2,437	This is a non-covered service because it is a routine/preventive exam or a diagnostic/screening procedure done in conjunction with a routine/preventive exam.
59	24,479	197	Processed based on multiple or concurrent procedure rules. (For example multiple surgery or diagnostic imaging, concurrent anesthesia.)
102	491	41	Major Medical Adjustment.

© 2024 Hoagland A et al. *JAMA Network Open.*

107	139	24	The related or qualifying claim/service was not identified on this claim.
111	15	1	Not covered unless the provider accepts assignment.
125	92	6	Submission/billing error(s).
129	5,263	842	Prior processing information appears incorrect.
140	66	1	Patient/Insured health identification number and name do not match.
146	176	40	Diagnosis was invalid for the date(s) of service reported.
147	105	15	Provider contracted/negotiated rate expired or not on file.
148	29	5	Information from another provider was not provided or was insufficient/incomplete.
163	681	31	Attachment/other documentation referenced on the claim was not received.
164	120	60	Attachment/other documentation referenced on the claim was not received in a timely fashion.
181	117	38	Procedure code was invalid on the date of service.
182	152	46	Procedure modifier was invalid on the date of service.
183	16	2	The referring provider is not eligible to refer the service billed.
185	1,224	17	The rendering provider is not eligible to perform the service billed.
189	39	13	'Not otherwise classified' or 'unlisted' procedure code (CPT/HCPCS) was billed when there is a specific procedure code for this procedure/service
206	439	59	National Provider Identifier – missing.
208	242	39	National Provider Identifier – Not matched.
210	74	18	Payment adjusted because pre-certification/authorization not received in a timely fashion
222	2,534	141	Exceeds the contracted maximum number of hours/days/units by this provider for this period. This is not patient specific.
226	2,442	286	Information requested from the Billing/Rendering Provider was not provided or not provided timely or was insufficient/incomplete.
236	1,987	348	This procedure or procedure/modifier combination is not compatible with another procedure or procedure/ modifier combination provided on the same day according to the National Correct Coding Initiative or workers compensation state regulations/ fee schedule requirements.
243	484	151	Services not authorized by network/primary care providers.
250	126	8	The attachment/other documentation that was received was the incorrect attachment/document. The expected attachment/document is still missing.
251	294	32	The attachment/other documentation that was received was incomplete or deficient. The necessary information is still needed to process the claim.
252	13,923	1,130	An attachment/other documentation is required to adjudicate this claim/service.
B12	285	100	Services not documented in patients' medical records.
B14	511	21	Only one visit or consultation per physician per day is covered.
B16	1,608	87	'New Patient' qualifications were not met.
B20	3,127	9	Procedure/service was partially or fully furnished by another provider.

© 2024 Hoagland A et al. *JAMA Network Open.*

B23	13	1	Procedure billed is not authorized per your Clinical Laboratory Improvement Amendment (CLIA) proficiency test.
B7	4,489	49	This provider was not certified/eligible to be paid for this procedure/service on this date of service.

4. Coverage Lapses

Code	Frequency, All Claims	Frequency, Preventive Services Only	Description
26	1,976	262	Expenses incurred prior to coverage.
27	11,974	864	Expenses incurred after coverage terminated.
31	3,104	757	Patient cannot be identified as our insured.
32	576	95	Our records indicate that this dependent is not an eligible dependent as defined.
33	868	37	Insured has no dependent coverage.
53	77	1	Services by an immediate relative or a member of the same household are not covered.
136	678	133	Failure to follow prior payer's coverage rules. (Use only with Group Code OA)
141	27	3	Claim spans eligible and ineligible periods of coverage.
166	34	5	These services were submitted after this payer's responsibility for processing claims under this plan ended.
200	1,283	497	Expenses incurred during lapse in coverage
239	4	3	Claim spans eligible and ineligible periods of coverage. Rebill separate claims.
106	29	2	Patient payment option/election not in effect.

5. Other Denials

Code	Frequency, All Claims	Frequency, Preventive Services Only	Description
60	2	1	Charges for outpatient services are not covered when performed within a period of time prior to or after inpatient services.
A1	148,548	745	Claim/Service denied.
B9	200	14	Patient is enrolled in a Hospice.

Note: Table frequencies are calculated at the date-of-service level and indicate the number of observed denial codes among all denied claims (column 2; n=407,731) and among only denied preventive claims (column 3; n=33,564). Note that each denied claim may have more than one denial code attached to it.

© 2024 Hoagland A et al. *JAMA Network Open.*

Table 3: Conditional Patients' Financial Responsibility for Denied Preventive Claims

	N Claims	% > 0	Min	Median	Mean	Max	SD
**Patient Groups**							
All Patients	61,785	92.85%	$0	$385	$1,395	$243,747	$5,612
Household Income ($000s)							
<$30	10,539	93.25%	$0	$412	$1,411	$196,514	$5,715
$30-49	6,951	93.58%	$0	$390	$1,654	$139,643	$6,374
$50-74	8,184	92.74%	$0	$354	$1,389	$243,747	$5,928
$75-99	7.281	92.39%	$0	$364	$1,383	$242,675	$5,609
>=$100	11,588	92.98%	$0	$365	$1,426	$240,493	$5,968
Patient Education							
High School or Less	14,860	93.63%	$0	$384	$1,397	$196,514	$5,672
Some College	19,515	92.43%	$0	$364	$1,441	$243,747	$5,856
Associate degree or Higher	9,694	93.12%	$0	$399	$1,485	$242,675	$6239
Race and Ethnicity							
Non-Hispanic White	29,765	92.85%	$0	$357	$1,467	$243,747	$6,104
Non-Hispanic Black	7,658	93.24%	$0	$390	$1,632	$242,675	$6,396
Hispanic	4,971	93.34%	$0	$464	$1,161	$196,514	$4,708
Asian	888	94.03%	$0	$522	$1,301	$46,032	$4,121
Other	651	92.78%	$0	$391	$1,084	$31,912	$2858

Notes: Distribution of unpaid portion of denied claims for preventive services, as defined using eTable 1. Claims include preventive services as well as any additional services consumed by the patient at the same provider on a given day; claims are then aggregated to the patient-provider-service day level. Unpaid portion of claims is measured as the total charges for the denied claim minus any amount paid by the plan and are adjusted to be in 2024 USD.

© 2024 Hoagland A et al. *JAMA Network Open.*

Table 4: Robustness of Regression Results to Individual-Specific Random Effects

	Any Claim Denial, OR [95% CI]			Specific Benefit Denial, OR [95% CI]			Billing Error Denial, OR [95% CI]		
	Primary Specification	First Service Only	50% Sample + RE	Primary Specification	First Service Only	50% Sample + RE	Primary Specification	First Service Only	50% Sample + RE
Patient Household Income (reference group: >$100,00 annually)									
<$30,000	1.430***	1.512***	1.751***	1.533***	1.609***	1.899***	1.160***	1.236***	1.277***
	(1.366 - 1.497)	(1.427 - 1.602)	(1.586 - 1.933)	(1.440 - 1.633)	(1.481 - 1.748)	(1.649 - 2.187)	(1.076 - 1.250)	(1.126 - 1.356)	(1.110 - 1.469)
$30,000–$49,999	1.264***	1.355***	1.424***	1.281***	1.410***	1.508***	1.103**	1.141***	1.124
	(1.204 - 1.327)	(1.274 - 1.442)	(1.281 - 1.583)	(1.196 - 1.371)	(1.290 - 1.541)	(1.297 - 1.755)	(1.019 - 1.194)	(1.034 - 1.259)	(0.969 - 1.305)
$50,000–$74,999	1.161***	1.213***	1.280***	1.245***	1.285***	1.415***	0.995	1.057	1.054
	(1.109 - 1.216)	(1.144 - 1.287)	(1.160 - 1.412)	(1.169 - 1.326)	(1.182 - 1.396)	(1.232 - 1.626)	(0.922 - 1.074)	(0.962 - 1.162)	(0.917 - 1.213)
$75,000–$99,999	1.109***	1.136***	1.157***	1.195***	1.204***	1.292***	0.990	1.038	1.003
	(1.058 - 1.162)	(1.071 - 1.206)	(1.048 - 1.277)	(1.122 - 1.273)	(1.108 - 1.309)	(1.125 - 1.484)	(0.915 - 1.071)	(0.943 - 1.143)	(0.870 - 1.157)
Patient Education (reference group: Associate degree or higher)									
<= High School	0.992	1.000	0.982	0.960	1.000	0.935	1.039	1.015	1.036
	(0.949 - 1.037)	(0.945 - 1.059)	(0.892 - 1.082)	(0.902 - 1.020)	(0.922 - 1.083)	(0.815 - 1.073)	(0.965 - 1.118)	(0.926 - 1.111)	(0.902 - 1.190)
Some College	0.958**	0.948**	0.925*	0.904***	0.893***	0.866**	1.040	1.006	1.017

© 2024 Hoagland A et al. *JAMA Network Open.*

	(0.921 - 0.996)	(0.902 - 0.996)	(0.851 - 1.005)	(0.857 - 0.952)	(0.833 - 0.957)	(0.770 - 0.972)	(0.973 - 1.111)	(0.927 - 1.090)	(0.901 - 1.147)
Patient Race/Ethnicity (reference group: Non-Hispanic White patients)									
Asian	1.537***	1.430***	1.805***	1.939***	1.749***	2.223***	1.143	1.166	1.327*
	(1.392 - 1.697)	(1.258 - 1.625)	(1.426 - 2.283)	(1.707 - 2.203)	(1.472 - 2.078)	(1.606 - 3.076)	(0.969 - 1.347)	(0.954 - 1.426)	(0.953 - 1.849)
Hispanic	1.158***	1.214***	1.313***	1.302***	1.363***	1.458***	1.015	1.092*	1.151*
	(1.101 - 1.218)	(1.139 - 1.293)	(1.171 - 1.471)	(1.214 - 1.397)	(1.247 - 1.491)	(1.241 - 1.714)	(0.938 - 1.098)	(0.991 - 1.203)	(0.984 - 1.345)
Non-Hispanic Black	1.191***	1.148***	1.178***	1.214***	1.129***	1.203***	1.147***	1.157***	1.085**
	(1.145 - 1.239)	(1.090 - 1.209)	(1.076 - 1.290)	(1.147 - 1.285)	(1.046 - 1.220)	(1.056 - 1.371)	(1.077 - 1.222)	(1.068 - 1.253)	(1.031 - 1.229)
Other	0.892*	0.917	0.756**	0.797**	0.749**	0.739	0.953	1.001	0.749
	(0.793 - 1.004)	(0.794 - 1.059)	(0.580 - 0.985)	(0.667 - 0.954)	(0.594 - 0.945)	(0.502 - 1.088)	(0.801 - 1.133)	(0.816 - 1.228)	(0.519 - 1.081)

Table Notes: Table presents regression coefficients for each of the three models discussed in the Methods section of the main text, with alternative specifications showing robustness of results after accommodating potentially correlated claims for individuals with multiple encounters. “Primary specification” refers to the main regression presented in Table 2; “First Service Only” subsets the data by including only the first claim per individual in the regression; and “50% Sample + RE” uses a 50% random sample of individuals in the data for regression, and adds an individual-specific random effect to regression adjustment.

© 2024 Hoagland A et al. *JAMA Network Open.*

eFigure 1: Overall Denial Rates (compare to Figure 1 in the main text)

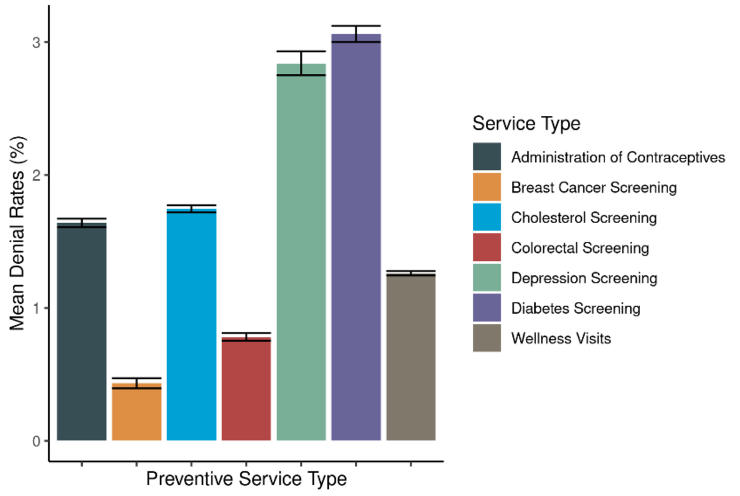

(a) All Services, by Type

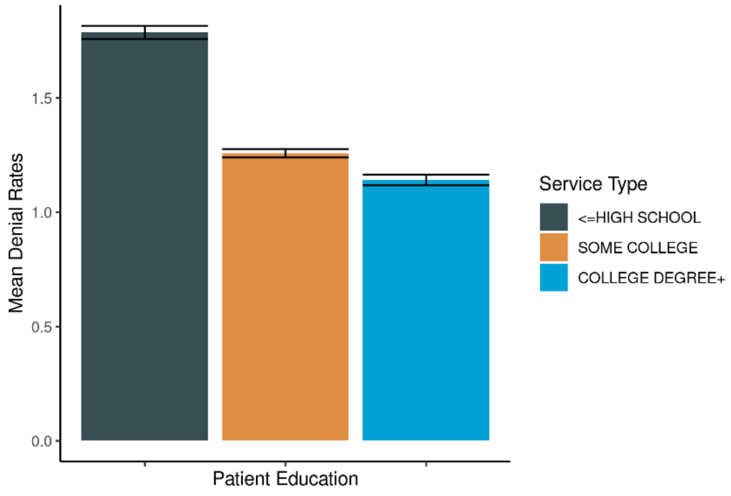

(b) Patient Education

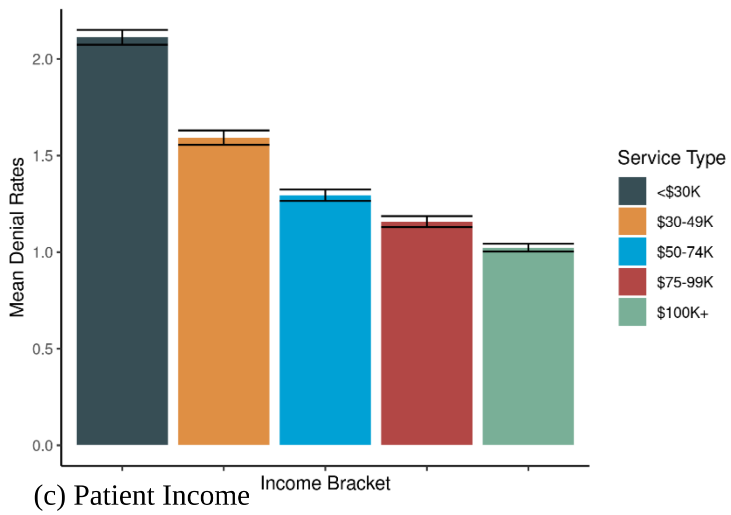

(c) Patient Income

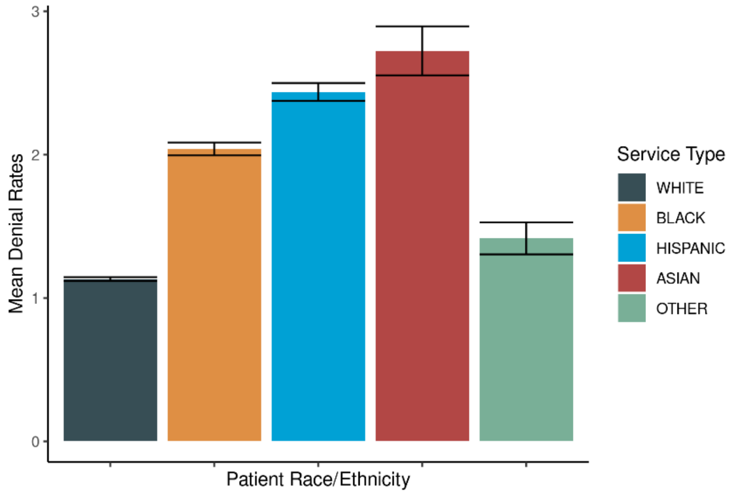

(d) Patient Race and Ethnicity

© 2024 Hoagland A et al. *JAMA Network Open.*

eFigure 2: Robustness of Inclusion of All Same-Day Services

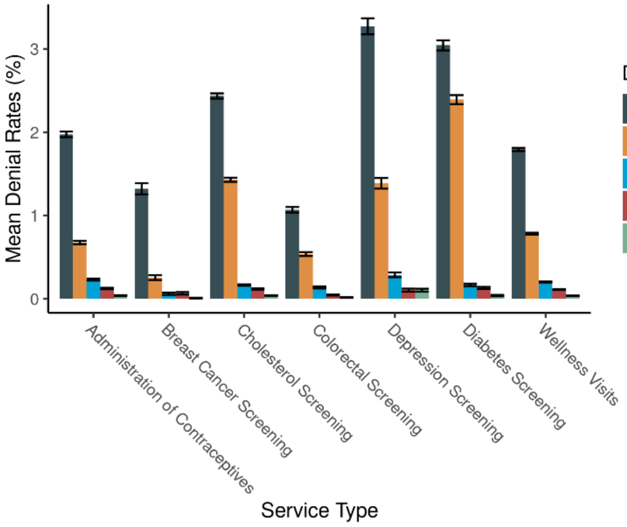

(a) Service Type

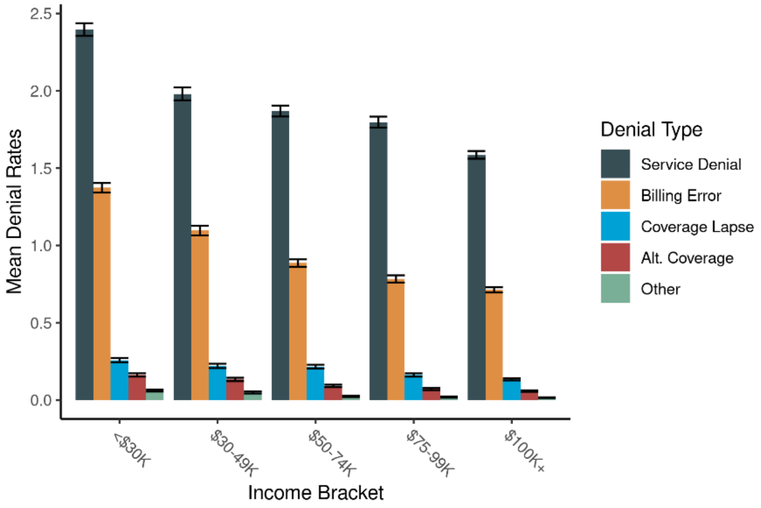

(b) Household Income Quintile

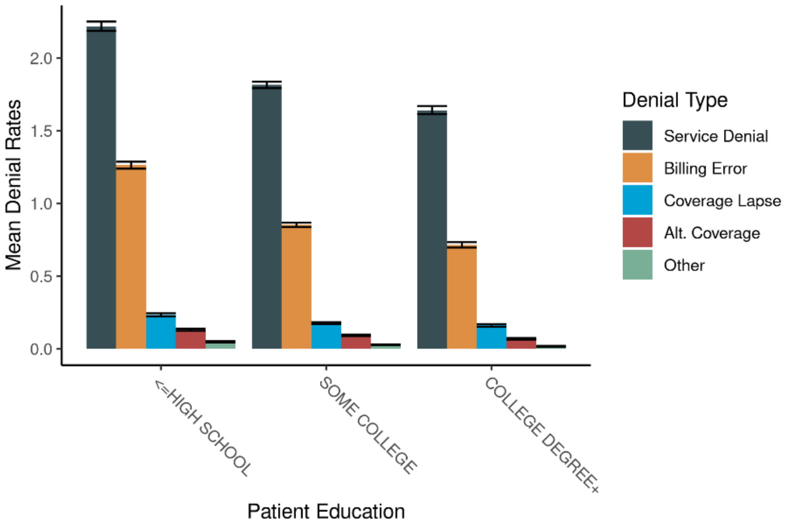

(c) Patient Education

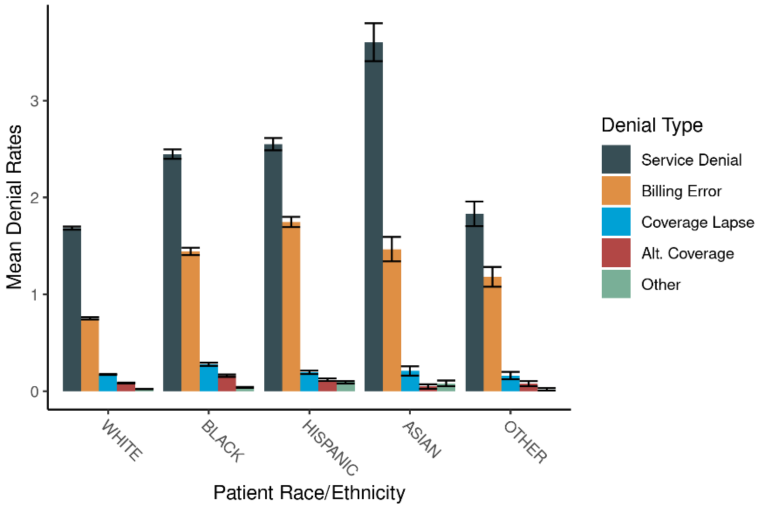

(d) Patient Race and Ethnicity

© 2024 Hoagland A et al. *JAMA Network Open.*

eFigure 3: Robustness of Exclusion of Wellness Visits

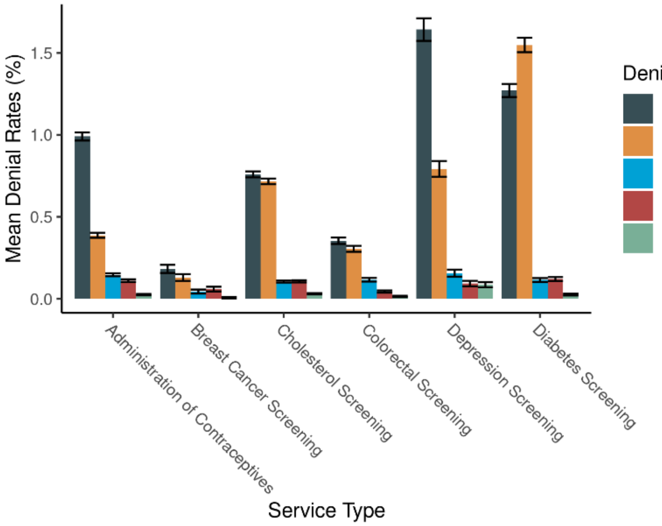

(d) Service Type

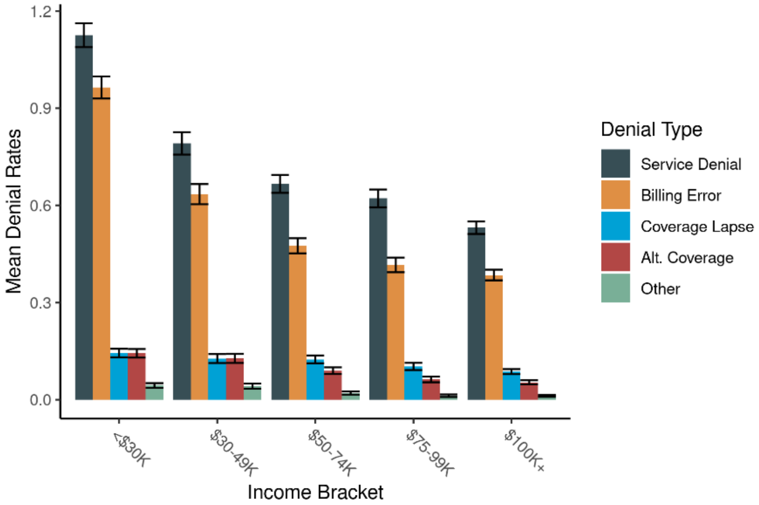

(a) Household Income Quintile

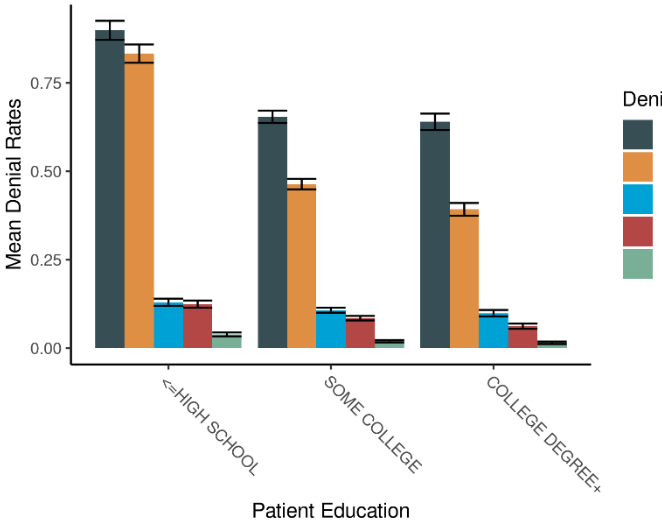

(b) Patient Education

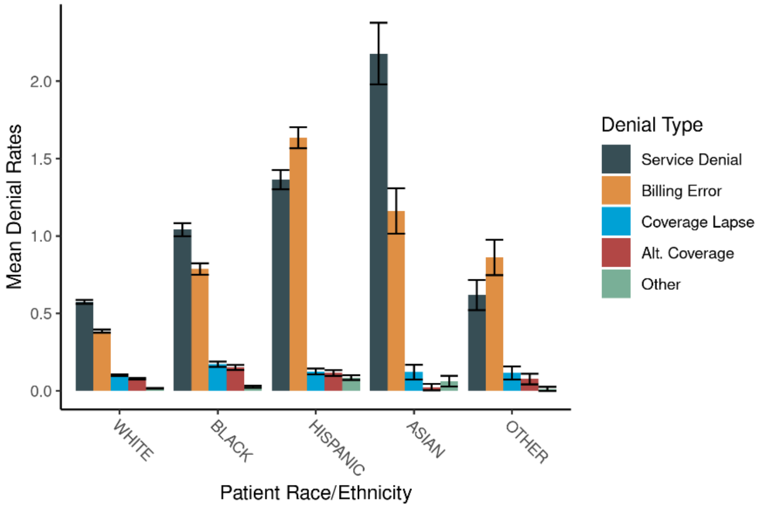

(c) Patient Race and Ethnicity

© 2024 Hoagland A et al. *JAMA Network Open.*
