# Supplementary material for: Social Determinants of Health and Insurance Claim Denials for Preventive Care
Source: JAMA Netw Open. 2024 Sep 18;7(9):e2433316. doi: 10.1001/jamanetworkopen.2024.33316 (PMC11411384; doi:10.1001/jamanetworkopen.2024.33316)
Supplement: Supplement 2. — Data Sharing Statement [file jamanetwopen-e2433316-s002.pdf]

Data Sharing Statement

Hoagland. Social Determinants of Health and Insurance Claim Denials for Preventive Care. *JAMA Netw Open*. Published September 18, 2024. doi:10.1001/jamanetworkopen.2024.33316

Data
Data available: No

Additional Information
Explanation for why data not available: Data is proprietary to Symphony Health Solutions and will not be shared by the authors.
